# Transient thrombocytopenia in a cat following G‐CSF treatment

**DOI:** 10.1002/vms3.706

**Published:** 2021-12-29

**Authors:** Kyeong‐Bo Kim, Ju‐Hyun An, Jeong‐Hwa Lee, Su‐Min Park, Hyung Kyu Chae, Woo‐Jin Song, Hwa‐Young Youn

**Affiliations:** ^1^ Laboratory of Veterinary Internal Medicine, Department of Veterinary Clinical Science, College of Veterinary Medicine and Research Institute for Veterinary Science Seoul National University Seoul Republic of Korea; ^2^ Department of Veterinary Internal Medicine College of Veterinary Medicine and Research Institute of Veterinary Research Jeju National University Jeju Korea

**Keywords:** cat, granulocyte colony‐stimulating factor, thrombocytopenia

## Abstract

A 4‐year‐old, castrated male, Russian blue cat with idiopathic epilepsy was diagnosed with neutropenia. The neutropenia was classified as idiopathic after blood tests and abdominal imaging did not reveal an infectious, inflammatory or neoplastic aetiology. As a treatment trial for idiopathic neutropenia, the cat was administered granulocyte colony‐stimulating factor by subcutaneous injection once daily for 3 days. Two weeks after completion of granulocyte colony‐stimulating factor therapy, the cat developed severe thrombocytopenia, with the granulocyte colony‐stimulating factor therapy considered to be the most likely cause. No treatment was initiated, and the thrombocytopenia had resolved spontaneously by 2 weeks after diagnosis. This is the first reported case of transient severe thrombocytopenia in a cat following granulocyte colony‐stimulating factor treatment.

## Graphical abstract

This is the first reported case of transient severe thrombocytopenia in a cat caused by granulocyte colony‐stimulating factor.

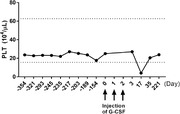

## 1 INTRODUCTION

As a therapeutic tool, human recombinant granulocyte colony stimulating factor (G‐CSF) exogenously stimulates haematopoietic precursors to increase the number of circulating neutrophils (Ferran et al., 2006). Common side effects of G‐CSF include bone and muscle pain, headache, fever and inflammation at the injection site. Serious complications include thrombosis, allergic reactions and spleen rupture (D'Souza et al., 2008). Thrombocytopenia has been reported to occur when G‐CSF is used as a therapeutic agent (Kovacic et al., 2007). However, there are no reports of thrombocytopenia after G‐CSF treatment in feline medicine. This is the first case report to describe transient thrombocytopenia in a cat following G‐CSF therapy.

## 2 CASE DESCRIPTION

A 4‐year‐old, castrated male, Russian blue cat with idiopathic epilepsy was managed with phenobarbital, zonisamide and levetiracetam. Five months after he stopped taking those drugs, leukopenia and neutropenia (white blood cells [WBC], 3940/μl, reference interval, 6300–19,600/μl; neutrophils, 1339/μl, reference interval, 3900–8000/μl; Advia 2120i Hematology system, Siemens Healthineers, Germany) was identified without clinical signs, such as dyspnoea, fever or lethargy. Serum biochemical examination and abdominal ultrasonography did not reveal any significant findings. Feline serum amyloid A concentration was within normal limits. The cat tested negative for feline leukaemia virus (FeLV), feline immunodeficiency virus (FIV) and feline parvo virus (FPV) using combination test kits, and negative for FeLV and FIV by real‐time PCR. Antinuclear antibody testing, performed to exclude autoimmune disorders, was negative. Evidence of other potential aetiologies, such as infectious, inflammatory or neoplastic disease, was not identified.

To treat the neutropenia, the cat was administered 5 μg/kg of recombinant human G‐CSF (Leukokine Inj 300; CJ Healthcare, Korea), once daily for 3 days by subcutaneous injection (Felix et al., 2005). Prior to G‐CSF therapy, apart from neutropenia, the complete blood count results were within normal limits [haemoglobin, 14.4 g/dl, reference interval, 8.1–14.2 g/dl; packed cell volume (PCV), 41.8 %, reference interval, 27.7–46.8 %; platelets (PLT), 250,000/μl, reference interval, 156,000–626,000/μl]. Physical examination revealed a rectal temperature of 38.3°C, heart rate (HR) of 228 beats per minute (bpm), respiratory rate (RR) of 28 per minute, systolic blood pressure (SBP) of 150 mmHg, but was otherwise unremarkable. After the 3‐day course of G‐CSF, the cat's WBC count had increased to 7660/μl, and the absolute neutrophil count was 4442/μl. Other cell lines were within their reference intervals (haemoglobin, 14.4 g/dl; PCV, 39.7 %; PLT, 271,000/μl). No abnormality was identified on physical examination (temperature, 38.3°C; HR, 186 bpm; RR, 48/min; SBP, 140 mm Hg). However, at day 15 after completion of G‐CSF treatment, the PLT count was 40,000/μl. For a more accurate evaluation, we analysed a manual blood smear. There was no evidence of platelet clumping and the number of platelets was found to be <2 cells/ 400× high power field in the blood smear. There was no evidence of bleeding on physical examination, and the PCV was similar to previous results. It would seem that the administration of G‐CSF was most likely because thrombocytopenia had been reported in humans, albeit rarely. Based on case reports in humans demonstrating spontaneous recovery from thrombocytopenia caused by G‐CSF, treatment was not initiated (Minelli et al., 2009; Wun, 1993). Two weeks after the diagnosis of thrombocytopenia, the cat's platelet count was 205,000/μl, within the reference interval.

## 3 DISCUSSION

G‐CSF is a glycoprotein that stimulates bone marrow production of granulocytes and stem cells and releases them into the bloodstream (Semerad et al., 2002). G‐CSF stimulates the survival, proliferation, differentiation and function of neutrophil precursors and mature neutrophils (Shirafuji et al., 1990). G‐CSF has been shown to be effective for the treatment of severe chronic neutropenia in humans in randomised controlled trials (Dale & Bolyard, 2017; Dale et al., 1993; Li et al., 2019).

Very few human patients have the adverse effects of G‐CSF sufficiently severe to warrant discontinuing treatment (Dale et al., 1993). Inflammation at the injection site and fever are common side effects. In addition, bone and muscle pain, splenomegaly, glomerulonephritis and capillary leak syndrome have been reported (Gavioli & Abrams, 2017; Ito et al., 2018; Rechner et al., 2003; Stroncek et al., 2003). Two healthy cats were treated experimentally with recombinant human G‐CSF for 21 days. Marked neutrophilia and variable lymphocytosis and monocytosis were recorded, but haematocrit and platelet counts did not change significantly (Fulton et al., 1991).

Several cases of G‐CSF causing thrombocytopenia have been reported in human medicine, although the mechanism is not known (Kovacic et al., 2007; Minelli et al., 2009). It has been suggested that the cause of thrombocytopenia in G‐CSF‐treated patients is related to under expression of proliferation‐related genes in megakaryocytes (Hernández et al., 2005), while another study postulated that injected G‐CSF responds to receptors present on platelets and affects mature platelets (Shimoda et al., 1993). Li et al. (2019) demonstrated that G‐CSF inhibited the differentiation of common myeloid progenitors into megakaryotic erythroid progenitors and subsequently decreased megakaryocyte and platelet production. Despite these hypotheses, the mechanism by which G‐CSF induces thrombocytopenia has not been fully established.

The time required for megakaryocyte maturation and release of platelets is approximately 3–5 days (Gant et al., 2020). Additionally, the life span of platelets is 5–10 days (Stockham & Scott, 2008). In this case, although platelet counts were not measured daily, thrombocytopenia was first detected 15 days after the last injection of G‐CSF. In addition, to determine whether thrombocytopenia induced by G‐CSF is a periodic pattern, platelet counts were checked 12 months before G‐CSF injection and 7 months after G‐CSF injection, and no values lower than the normal range were found (Additional file 1). We consider thrombocytopenia seen in this case to be most likely secondary to G‐CSF treatment considering our knowledge of the expected timeline of megakaryocyte maturation, platelet release and platelet lifespan as well as the transient nature of the thrombocytopenia and the lack of any other probable cause. Furthermore, development of G‐CSF‐induced thrombocytopenia in this case was similar to reports in human medicine (Minelli et al., 2009; Wun, 1993).

In this case, no evidence of other potential causes for neutropenia, such as infectious, inflammatory or neoplastic disease, was found (Dale & Bolyard, 2017). Although this patient was previously administered anticonvulsants for management of idiopathic epilepsy, including phenobarbital, zonisamide and levetiracetam, which have the potential to produce neutropenia, none of these drugs had been administered in the 5 months preceding G‐CSF treatment. Neutropenia lasting for at least for 3 months and not attributable to drugs or infectious, inflammatory, autoimmune or malignant cause is called chronic idiopathic neutropenia (Dale & Bolyard, 2017). In addition, diagnosis depends on the physical exam and more extensive laboratory testing to exclude other diseases. In this case, there were no underlying disease processes found that would induce neutropenia, nor were there clinical signs consistent with febrile neutropenia. We consider idiopathic neutropenia the most likely underlying diagnosis, although ideally bone marrow testing would have been performed as part of the diagnostic work‐up.

Treatment for G‐CSF‐induced thrombocytopenia had not been clearly established in feline medicine. In a single human case report, severe thrombocytopenia associated with G‐CSF treatment spontaneously resolved within 65 days (Wun, 1993). Another study reported that in a healthy male bone marrow donor who developed thrombocytopenia after G‐CSF treatment, platelet levels rebounded to normal 9 days after stopping G‐CSF (Minelli et al., 2009). Based on these reports, it was decided not to initiate treatment for thrombocytopenia in this cat and the platelet count was within the reference interval 4 weeks after discontinuation of G‐CSF. This case report is an important addition to the literature and adds to the limited body of knowledge about the application and safety of human G‐CSF treatment in cats.

## 4 CONCLUSION

This is the first reported case of thrombocytopenia following G‐CSF treatment in a cat. These results indicate that the potential for thrombocytopenia should be considered when using G‐CSF for treatment of neutropenia in feline medicine.

## ETHICS STATEMENT

The authors confirm that the ethical policies of the journal, as noted on the journal's author guidelines page, have been adhered to. No ethical approval was required as this is a case report with no original research data.

## AUTHOR CONTRIBUTIONS

Kyeong‐bo Kim & Ju‐Hyun An: conceptualisation; investigation; resources; writing‐original draft. Jeong‐Hwa Lee, Su‐Min Park, Hyung‐Kyu Chae: investigation; validation.

Woo‐Jin Song & Hwa‐Young Youn: investigation; validation; writing‐review & editing.

## CONFLICT OF INTEREST

The authors declared no potential conflicts of interest with respect to the research, authorship and/or publication of this article.

### PEER REVIEW

The peer review history for this article is available at https://publons.com/publon/10.1002/vms3.706

## Data Availability

Data openly available in a public repository that issues datasets.

## 1

**Additional file 1. Platelet count before and after human G‐CSF treatment**. Graph showing platelet count values from the time of admission with G‐CSF to the period before and after treatment.
